# Multifunctional Nanoregulator Reshapes Immune Microenvironment and Enhances Immune Memory for Tumor Immunotherapy

**DOI:** 10.1002/advs.201900037

**Published:** 2019-06-17

**Authors:** Meng Yu, Xiaohui Duan, Yujun Cai, Fang Zhang, Shuqi Jiang, Shisong Han, Jun Shen, Xintao Shuai

**Affiliations:** ^1^ Department of Radiology Sun Yat‐sen Memorial Hospital of Sun Yat‐sen University Guangzhou 510120 China; ^2^ PCFM Lab of Ministry of Education School of Material Science and Engineering Sun Yat‐Sen University Guangzhou 510275 China; ^3^ Guangdong Provincial Key Laboratory of Malignant Tumor Epigenetics and Gene Regulation Sun Yat‐sen Memorial Hospital Sun Yat‐Sen University Guangzhou 510120 China

**Keywords:** hypoxia, myeloid‐derived suppressor cells, PI3Kγ inhibitor, triggered release, tumor immune microenvironment

## Abstract

Hypoxia leads to up‐regulation of PD‐L1 and decreases T lymphocyte infiltration, thus boosting immunotherapeutic resistance of tumors. Moreover, tumor‐infiltrating myeloid cells such as myeloid‐derived suppressor cells (MDSCs) correlate with potent immune suppressive activity and resistance to the immune checkpoint blocking (ICB) in tumor sites. Here, a multifunctional nanoregulator incorporating MnO_2_ particles and small molecular IPI549 is developed, which can reshape the tumor immune microenvironment (TIME) to unleash the immune system. The intravenously administered nanoregulator effectively accumulates in tumor sites to alleviate hypoxia via oxygen‐generating reduction of MnO_2_ and to inhibit PI3Kγ on MDSCs via IPI549 release in the tumor microenvironment (TME), which results in concurrent downregulation of PD‐L1 expression, polarization of tumor associated macrophages (TAMs) toward pro‐inflammatory M1‐like phenotype (tumor‐suppressive), enhanced infiltration of CD4^+^ helper T lymphocytes (Th cells), and cytotoxic CD8^+^ T lymphocytes (Tc cells), and suppressed infiltration of regulatory T lymphocytes (T_reg_ cells) for effective tumor immunotherapy. Furthermore, the local generation of Mn^2+^ in TME allows tumor‐specific magnetic resonance imaging (MRI). More excitingly, the nanoregulator‐reshaped TIME is effectively reserved due to the synergistic effect of hypoxia alleviation and MDSC PI3Kγ inhibition, leading to remarkable post‐medication inhibition of tumor re‐growth and metastasis in an animal study.

## 1 Introduction

Cancer is the leading cause of death worldwide, with over 10 million new cases every year in Europe and the US.^1^ Traditional tumor treatment strategies, including surgery, chemotherapy, radiotherapy, and phototherapy, mostly focus on tumor cell killing, which often fail due to the complicated tumor recurrence and progression process involving multiple cell types and acquired chemo‐/radio‐resistance of cancer cells. Unlike cancer cells, the stromal cells in tumor microenvironment (TME) possessing more stable genotypes have proven suitable as therapeutic targets as well, which provides the basis for emerging tumor immunotherapy. For instances, Clever^2^ and Sitkovsky^3^ developed approaches via regulating the functions of T cells, while Medzhitov focused on tumor‐associated macrophages (TAMs).^4^ Immunotherapeutic drugs showed strong effect on tumor growth inhibition by activating the human immune system. Unfortunately, tumor may develop various pathways to escape the immunotherapy, chief among them being the activation of immune checkpoints against immune system attack. Therefore, immune checkpoint blocking (ICB) antibodies against cytotoxic T lymphocyte‐associated protein 4 (CTLA‐4) or programmed cell death protein 1/programmed cell death‐ligand 1 (PD‐1/PD‐L1) have attracted tremendous attention owing to their remarkable therapeutic effects in treatments of various cancers.^5^ Yet, growing evidences have suggested that myeloid‐derived suppressor cells (MDSCs) (CD11b^+^) constitute the majority of CD45^+^ leukocytes, which correlate with the reduced infiltration and cytolytic function of CD8^+^ T cells leading to resistance to immune checkpoint inhibitors.^6^, ^7^, ^8^ Infiltration of MDSCs protects tumor cells from immune‐mediated killing, remodels tumor immune microenvironment (TIME), establishes re‐metastatic niche, and promotes epithelial‐to‐mesenchymal transition (EMT).^9^ In addition, MDSCs were capable of producing VEGF, bFGF, VEGF‐analogue Bv8, and MMP9 to mediate tumor neoangiogenesis and metastasis.^10^, ^11^, ^12^ On the other hand, unlike mature myeloid cells such as macrophages, DCs and neutrophils, MDSCs possess polarization potentials toward different TAMs with M1‐ and M2‐like phenotypes, respectively.^13^ Although the classic pro‐inflammatory M1 phenotype exerted antiproliferative and cytotoxic activities, the M2‐skewed immunosuppression prevailed in TME.^14^ Kaneda et al.^15^ and other researchers^16^, ^17^ have shown that gamma isoform of phosphoinositide 3‐kinase (PI3Kγ) highly expressed in MDSCs can promote their tumor migration and reduce production of inflammatory mediators. Selective inhibition of PI3Kγ reshaped TIME by switching macrophages from the immunosuppressive M2‐like phenotype to the pro‐inflammatory M1‐like state, thus restoring sensitivity to ICB and promoting tumor regression via synergistic activity with cytotoxic T cells.^8^ A small molecular PI3Kγ inhibitor, IPI549, has shown not only a favorable pharmacokinetic profile that allows potent and selective inhibition of PI3K‐γ in vivo^8^ but also excellent oral bioavailability, low clearance, and good biosafety in animal studies. More excitingly, severe side effects have not been reported for a phase 1 clinical trial of IPI‐549 (NCT02637531), which further pushed forward a phase 2 clinical trial (NCT03795610).

On the other hand, hypoxia of cancerous tissues has been confirmed to affect tumor immunotherapy. The extracellular adenosine enriched in hypoxic cancerous tissue signals via A2A and A2B adenosine receptors expressed on antitumor CD8^+^ T cells and NK cells to result in immunosuppression.^18^, ^19^ Moreover, hypoxia‐inducible factor‐1α (HIF‐1α) selectively up‐regulates PD‐L1 by binding to the hypoxia response elements (HRE) in the PD‐L1 proximal promoter.^20^ In recent studies, the use of respiratory hyperoxia (60% oxygen) as an antiadenosinergic treatment improved cancer immunotherapy by unleashing antitumor T and NK cells.^21^, ^22^ So far, the O_2_ supply based on high reactivity of MnO_2_ with H_2_O_2_ in tumor acidic environment has proven to be advantageous over other O_2_‐supplying approaches using perfluorocarbon,^23^ catalase,^24^ carbon nitride,^25^ and high‐Z elements.^26^ In situ production of O_2_ effectively relieved tumor hypoxia by targeted delivery of MnO_2_.^27^, ^28^, ^29^, ^30^

It is well known that the codelivery of different therapeutic agents boosts their synergistic anticancer effect via multiple target‐based combination therapy.^31^, ^32^, ^33^ This strategy can be utilized to develop a multifunctional nanoregulator which can release O_2_ for hypoxia relief and IPI549 for PI3Kγ inhibition on MDSCs in response to TME. By this means, the tumor‐specific production of O_2_ may reduce hypoxia‐related cytokines (e.g., HIF‐1α) and PD‐L1 to unleash immune‐promoting T cells, which may assist the immunotherapeutic effect of PI3Kγ inhibition in tumor sites. In addition to producing O_2_, MnO_2_ reacts with H_2_O_2_ and acid in tumor tissue to generate Mn^2+^,^28^ while Mn^2+^ is a potent *T*_1_‐weighted contrast agent for MRI. Therefore, unlike the clinically used Gd‐based *T*_1_ agents incapable of tumor‐sensing, a TME‐triggered MRI can be achieved by tumor‐targeted MnO_2_ delivery, which is favorable for significantly improving the specificity of MRI technique in detecting solid tumors.^34^

**Scheme 1:**
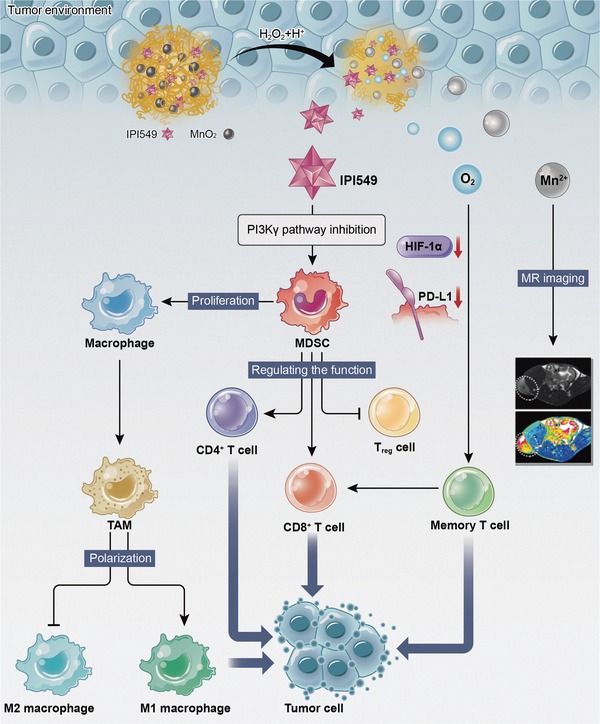
Schematic illustration of BSA‐MnO_2_‐IPI549 nanoregulator as a stimulation‐responsive platform to reshape tumor immune microenvironment (TIME) for MRI‐guided immunotherapy.

Here, we describe a multifunctional TIME nanoregulator (TIME‐NR) incorporating MnO_2_ and a PI3Kγ inhibitor (IPI549) for MRI‐visible cancer immunotherapy via normalizing the hypoxic and immunosuppressive TME (**Scheme** 1). Bovine serum albumin (BSA) featuring excellent biocompatibility was used as drug carrier to encapsulate both MnO_2_ nanoparticles and IPI549, through a simple biomineralization process using MnCl_2_ in alkaline conditions.^35^, ^36^, ^37^ The thus obtained TME‐responsive TIME nanoregulator, BSA‐MnO_2_‐IPI549 (BMI), was expected to facilitate MRI‐visible tumor immunotherapy. First, MnO_2_ is highly reactive with H_2_O_2_ and acid enriched in tumor tissue to generate O_2_ and Mn^2+^, which would not only result in a modulation of hypoxic TME but also enable a highly sensitive *T*_1_‐weighted MRI. Second, the degradation of MnO_2_ as a major component would lead to nanoregulator collapse, triggering burst release of IPI549 to exert immunotherapeutic effect in tumor sites. In vitro and in vivo experiments were carried out to explore the potential of using this multifunctional nanoregulator to reshape TIME for effective tumor immunotherapy.

## 2 Results and Discussion

### 2.1 Physicochemical Properties of Nanoregulators

**Figure 1:**
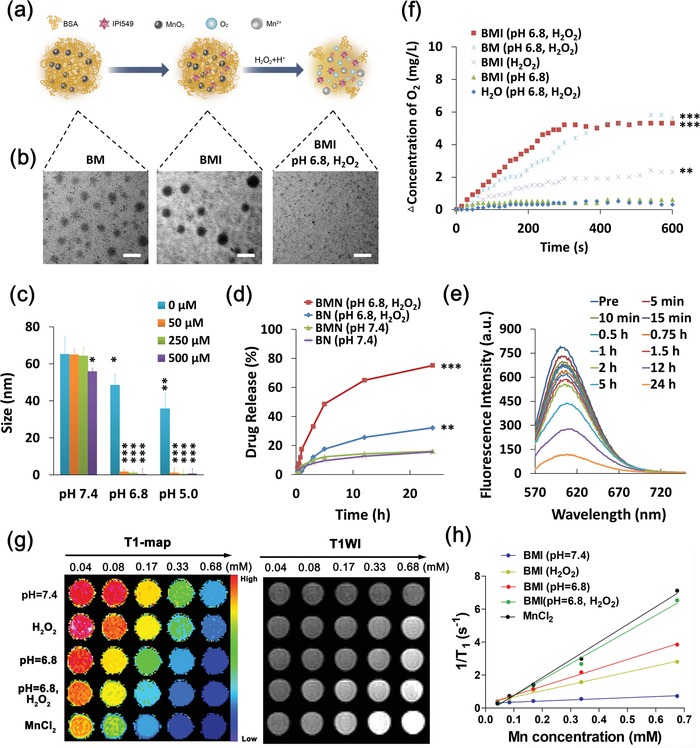
Tumor microenvironment (TME)–responsive drug release and hypoxia relief of MnO_2_‐embedded nanoparticles evaluated in vitro. a) A scheme showing the pH/H_2_O_2_ responsiveness of BMI. b) TEM images of BM, BMI, and BMI after triggered degradation under H_2_O_2_ and acidic conditions. Scale bar: 200 nm. c) The degradation behavior of BMI dispersed in aqueous solutions with different pH values and H_2_O_2_ concentrations measured by DLS (*n* = 3). d) The TME‐responsive drug release behavior of tested nanoparticles with or without MnO_2_ encapsulation measured by fluorescent spectrometry (*n* = 3). NR was encapsulated in nanoparticles as a substitute of hydrophobic IPI549. e) Change of fluorescence of NR‐encapsulated BMN in aqueous solution (50 × 10^−6^ m H_2_O_2_; pH 6.8) over the course of incubation (*n* = 3). f) O_2_ generation in solutions containing H_2_O_2_ (50 × 10^−6^ m) and MnO_2_‐embedded nanoparticles at pH 7.4 and pH 6.8 (*n* = 3). g) T1‐map and T1WI, h) longitudinal relaxivity (*r1*) of BMI. The magnetic relaxation properties of MnCl_2_ were measured as a positive control. *P < 0.05, **P < 0.01, ***P < 0.001.

The nanoparticles measuring about 65 nm in dynamic light scattering (DLS) regardless of IPI549 loading were well dispersed in aqueous solution and stable in serum‐containing medium over prolonged experimental time (Figure S1, Supporting Information). The drug‐free nanoparticle (BSA‐MnO_2_, BM) and drug‐loaded nanoregulator (BSA‐MnO_2_‐IPI549, BMI) both exhibited spherical morphology with uniform size distribution under TEM observation (**Figure** 1a,b).

It is known that the degradation of MnO_2_ could be triggered in acidic environment with enriched H_2_O_2_, which is a distinct feature for solid tumors.^30^, ^38^ Thus, we investigated the MnO_2_ reactivity of nanoparticles in various aqueous solutions. As MnO_2_ degradation inevitably led to disassembly of nanoparticles, the changes of particle size were monitored to indicate stimuli‐responsiveness (Figure 1c). The pH sensitivity appeared more critical in determining the pH/H_2_O_2_ dual responsiveness of both BM and BMI in aqueous solutions. At pH 7.4, BMI showed fairly constant particle size regardless of the presence of H_2_O_2_ up to 500 × 10^−6^ m. However, even in the absence of H_2_O_2_, pH decline to 6.8 still led to a clear decrease in particle size. Moreover, BMI seemed to fully disassemble at pH 6.8 and 5.0 even at a very low concentration of H_2_O_2_ (50 × 10^−6^ m), i.e., almost no size detectable by DLS. Meanwhile, the spherical nanoparticles measuring 65 nm for intact BMI was no longer observable in the TEM images (Figure 1a,b). Instead, a number of small pieces with irregular morphology and various sizes were seen, which further demonstrated the decomposition of BMI at low pH and enriched H_2_O_2_.

Nile red (NR), as a commonly applied analogue of hydrophobic drugs,^39^ was encapsulated to show the drug release behavior of nanoregulator. The release behavior was easily detectable because NR was fluorescent inside nanocarriers but nonfluorescent at free form because of its extremely low solubility in aqueous solution. The cumulative release behaviors of NR from NR/MnO_2_‐coloaded nanoparticle (BMN) and NR‐loaded/MnO_2_‐free nanoparticle (BN) were recorded at acidic (pH 6.8) and H_2_O_2_‐enriched (50 × 10^−6^ m) conditions (Figure 1d), which showed that BMN released NR much faster than BN due to the MnO_2_ decomposition. Release of NR from BMN reached above 50% within just 5 h and about 80% within 24 h, whereas release of NR from BN only reached 35% within 24 h. As shown in Figure 1e, the NR fluorescence of BMN solution decreased against time at 50 × 10^−6^ m H_2_O_2_ and pH 6.8, which was attributed to the precipitation of released NR out of the solution.^39^

The dissolved O_2_ levels of BMI and BM solutions were determined using a portable dissolved oxygen meter in order to assess the O_2_ generation of MnO_2_ in the presence of H^+^ and H_2_O_2_. As shown in Figure 1f, the MnO_2_‐containing nanoparticles generated O_2_ quickly due to concurrent stimulations of H^+^ (pH 6.8) and H_2_O_2_ (50 × 10^−6^ m), whereas the O_2_ concentration of nanoparticle‐free solution did not change under the same stimulation conditions. Notably, the nanoparticles exhibited no O_2_ generation at pH 6.8 but without H_2_O_2_. In contrast, the nanoparticles still displayed a low level of O_2_ generation under H_2_O_2_ stimulation alone, likely because MnO_2_ may catalyze H_2_O_2_ decomposition into H_2_O and O_2_.^40^

### 2.2 Magnetic Resonance Imaging Potentials

MnO_2_ have been found to be a novel tumor microenvironment‐triggerable MRI contrast agent.^40^, ^41^ It is stable under neutral and alkaline conditions with low *r*_1_ and *r*_2_ values. However, in TME featuring low pH (around 6.8) and rich H_2_O_2_ (about 50 × 10^−6^ m), MnO_2_ could be reduced into Mn^2+^ with five unpaired 3d electrons, which is easily accessible to surrounding water molecules to decrease the transverse and longitudinal relaxation time of protons. To evaluate the potential of BMI as a TME‐activated MRI contrast agent, longitudinal relaxivity (*r*_1_) and transverse relaxivity (*r*_2_) of BMI subjected to treatment of H_2_O_2_ (50 × 10^−6^ m) or/and acid (pH 6.8) in PBS were measured at 3.0 T field strength (Figure 1g,h and Figure S2, Supporting Information). The averaged *r*_1_ and *r*_2_ values of BMI at pH 7.4 without H_2_O_2_ treatment were 0.67 and 3.31 mM^−1^ s^−1^, respectively. Treatments at 50 × 10^−6^ m H_2_O_2_ (pH 7.4), pH 6.8 without H_2_O_2_, pH 6.8 plus 50 × 10^−6^ m H_2_O_2_ increased the averaged *r*_1_ value of BMI to 3.78, 5.43, and 9.76 mM^−1^ s^−1^, and the averaged *r*_2_ values of BMI to 13.88, 33.74, and 50.64 mM^−1^ s^−1^, respectively. The averaged *r*_1_ and *r*_2_ values of BMI at pH 6.8 plus 50 × 10^−6^ m H_2_O_2_ were approximate to that of the MnCl_2_ positive control (*r*_1_ = 10.33 ± 0.43 mM^−1^ s^−1^; *r*_2_ = 58.17 ± 2.13 mM^−1^ s^−1^). Notably, although its *r*_2_ value was lower than the commercialized Feridex (*r*_2_ = 108 mM^−1^ s^−1^), its r_1_ value at pH 6.8 plus 50 × 10^−6^ m H_2_O_2_ was much higher than that of the clinically used Gd‐DPTA (*r*_1_ = 3.5–5.5 mM^−1^ s^−1^),^41^ which strongly suggested that BMI mainly exhibits T1 contrast enhancement. Moreover, compared to the commercial MRI contrast agents without tumor specificity, the TME‐triggerable imaging capacity may endow BMI with a great potential to avoid false‐positive signals in MRI diagnosis of tumor.

### 2.3 In Vitro Cell Studies

**Figure 2:**
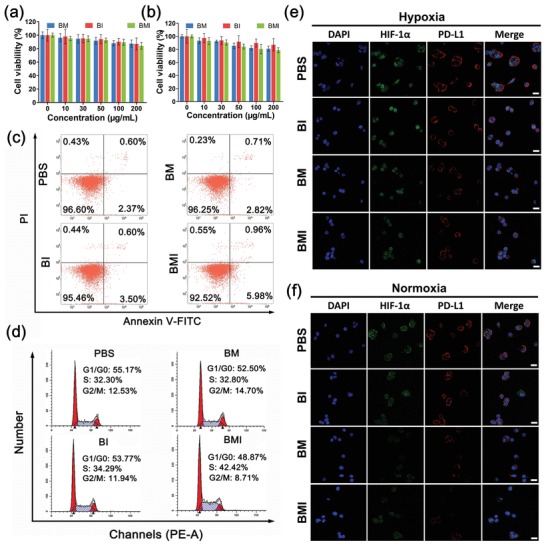
In vitro cytotoxicity and expression of hypoxia‐related biomarkers. Relative viabilities of a) 4T1 cells and b) HUVEC cells after incubation for 24 h with BM, BI, and BMI at different concentrations (*n* = 6). Flow cytometry assays of c) apoptotic rates and d) cell cycle distribution, and e,f) immunofluorescence analysis of HIF‐1α and PD‐L1 expression of 4T1 cells after incubation for 24 h with different formulations (200 µg mL^−1^). Scale bar: 50 µm.

The 4T1 cells (mouse breast cancer cells) and HUVECs (human umbilical vein endothelial cells) incubated with BM, BI, and BMI even at high concentrations up to 200 µM mL^−1^ showed almost no changes in viabilities, apoptosis rates, and cell cycle distributions as compared with the control cells (**Figure** 2a–d), indicating the low cytotoxicity of these nanoparticles in both cancer cells and normal cells. These results are reasonable in consideration of the high biocompatibilities of the two major components BSA and MnO_2_. It is worth noting that BSA, like the human albumin for paclitaxel delivery in clinical use, has drawn great attentions as a natural drug carrier material.^42^, ^43^ Our results evidenced that the TIME nanoregulator (BMI) carrying the PI3Kγ inhibitor IPI549 did not directly kill cancer cells.

It is well known that tumor cells overexpress the transmembrane protein ligand PD‐L1, which binds the PD‐1 receptors on T cells to decrease their tumor cell‐killing activity.^5^ Therefore, we first evaluated whether the nanoregulator was able to activate immune system by downregulating the PD‐L1 expression of cancer cells cultured under both hypoxic and normoxic conditions. As shown in Figure 2e,f and Figure S3 (Supporting Information), immunofluorescent staining and Western blotting assay showed that BI carrying IPI549 alone did not affect the PD‐L1 expression of 4T1 cells. On the contrary, cell treatments with the MnO_2_‐embedded nanoregulators, i.e., BM and BMI, both resulted in significant down‐regulation of PD‐L1 expression. Moreover, BMI appeared even more potent than BM in down‐regulating PD‐L1 expression. These results clearly indicated that MnO_2_ delivery played a key role in the PD‐L1 suppression in 4T1 cancer cells, while IPI549 could strengthen such effect only when it was codelivered with MnO_2_. The underlying reason for this phenomenon was not clear yet.

### 2.4 In Vivo Distribution of Nanoregulator

**Figure 3:**
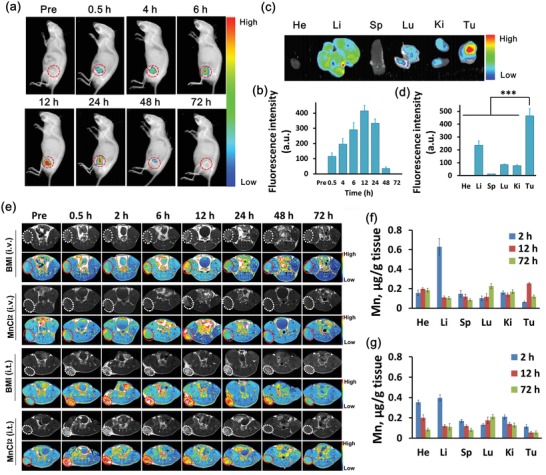
In vivo distribution and tumor microenvironment (TME)–responsive MR imaging of nanoregulator (BMI). a) Representative in vivo fluorescence imaging and b) relative fluorescence intensity of 4T1 tumor‐bearing mice intravenously injected with DiR‐labeled BMI (1 mg DiR kg^−1^) at different time points. c) Ex vivo fluorescent imaging and d) relative fluorescence intensity of DiR in major organs (heart, He; liver, Li; spleen, Sp; lung, Lu; kidney, Ki; tumor, Tu) at 12 h after injection. e) Representative in vivo serial MR images of subcutaneous 4T1 tumor‐bearing mice before and after intravenous injection (5 mg Mn kg^−1^), as well as before and after intratumoral injection of BMI or MnCl_2_ at different time points on T1WI. Distribution of Mn at 2, 12, and 72 h after IV injection of f) BMI or g) MnCl_2_ solution in 4T1 tumor models (*n* = 3). ***P < 0.001.

Fluorescence imaging experiments were performed to explore the in vivo distribution of BMI after tail vein injection. To make the nanoregulator visible in fluorescence imaging, 1,1′‐dioctadecyl‐3,3,3′,3′‐tetramethyl indotricarbocyanine iodide (DiR) was loaded into the BMI nanoparticles together with IPI549 (Figure S4, Supporting Information, Ex = 764 nm and Em = 780 nm). The in vivo fluorescence imaging showed that the DiR fluorescence intensity in tumor increased gradually until reaching the highest at 12 h after IV injection of DiR‐labeled BMI (**Figure** 3a,b; Figure S5, Supporting Information). After that, a gradual decay of tumor DiR fluorescence was observed. At 72 h after IV injection, tumor site showed no detectable DiR fluorescence, indicating an almost complete clearance. The BMI distribution in major organs was analyzed by ex vivo fluorescence imaging as well, which revealed a preferential accumulation in tumor (Figure 3c,d).

The development of MRI contrast agents responsive to the intrinsic physiological microenvironment (pH, redox, H_2_O_2_, enzyme, heat, etc.) and/or external stimulations (magnetic/electronic field, ultrasound, light, etc.) is attracting increasing attention in recent years.^28^, ^30^, ^40^, ^44^, ^45^ It could substantially increase the MRI contrast of target to background in a highly site‐specific manner, thus improving the imaging accuracy and sensitivity for early diagnosis and biochemical activity sensing.^46^, ^47^ Unlike conventional MRI contrast agents, the in vivo TME‐responsive MRI contrast agents may enable diseased area‐specific imaging via reducing false imaging due to nonspecific distribution of contrast agents. A series of MR T1WI examinations were performed herein to evaluate the potential of BMI as a TME‐responsive MRI contrast agent. After tail vein injection of BMI, the MRI signal intensity of tumor area increased gradually to reach the highest value at 12 h and then declined to the basal level within 72 h after injection. As Mn^2+^ rather than MnO_2_ was known to enhance MRI contrast,^28^ the above results also meant that the MnO_2_ delivered to tumor site was reduced to Mn^2+^ by H^+^ and H_2_O_2_ enriched therein. This notion was supported by MRI study of animals receiving direct intratumoral injections of BMI and MnCl_2_, respectively (Figure 3e; Figure S6, Supporting Information). At the same injection dose (5 mg Mn kg^−1^), both injections showed significant enhancement of the imaging signals at tumor site, which provided strong evidence that MnO_2_ was effectively converted to Mn^2+^ inside tumor for potent MR imaging. In comparison, the signal intensities increased much faster to reach a peak and then declined much faster as well when MnCl_2_ was injected, which was apparently due to the time‐consuming reduction of MnO_2_.

We further assessed the in vivo distribution of nanoregulator at three postinjection time points by determining Mn depositions in major organs and tumors with ICP‐MS using MnCl_2_ as a control (Figure 3f,g). After IV injection, MnCl_2_ showed tumor accumulation of 0.11, 0.18, and 0.05 µg Mn g^−1^ tissue at 2, 12, and 72 h respectively, indicating a quick tumor accumulation and a fast clearance. In contrast, the intratumoral Mn concentrations for BMI injection were 0.10, 0.26, and 0.07 µg Mn g^−1^ tissue at 2, 12, and 72 h, respectively, which clearly indicated a slow but much better tumor accumulation (0.26 µg g^−1^ tissue vs 0.18 µg g^−1^ tissue) owing to the tumor EPR effect.^48^ In addition, similar distributions of BMI and MnCl_2_ in major organs were detected. Nevertheless, BMI showed a higher level of liver accumulation but a lower level of heart accumulation than MnCl_2_ at 2 h after injection, which are in line with the well‐known hepatic metabolism of nanoparticles.^49^ Notably, the strong liver accumulation of BMI hardly provided enhancement to MRI signal intensity (Figure S7, Supporting Information). Because the stimuli (i.e., enriched H_2_O_2_ and H^+^) for MnO_2_ reaction to generate Mn^2+^ were absent in liver, only a slight and transient enhancement to the MRI signal intensity was detected due to the weak T1 contrast enhancement of MnO_2_ itself (Figure 1g,h). As MnO_2_ decomposition was required for IPI549 release (Figure 1d), our results implied that the nanoregulator would not impose strong side effects via altering the normal immune microenvironment of healthy tissue and organ where it accumulated in addition to tumor. The disassembly/decomposition inside tumor leading to the in situ IPI549/Mn^2+^ release is essential for the nanoregulator (BMI) to exert a tumor‐specific immunotherapeutic effect.

### 2.5 Immunotherapeutic Effects of Nanoregulators

**Figure 4:**
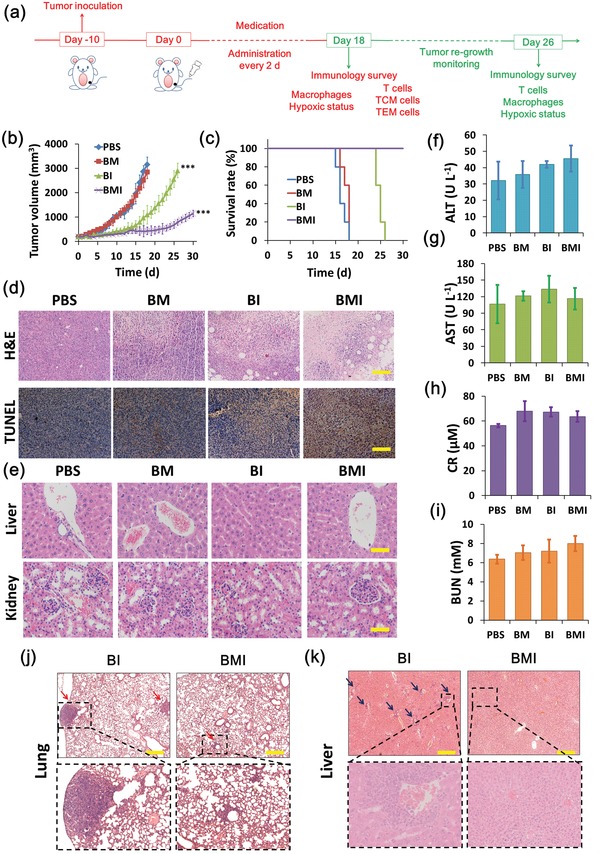
In vivo immunotherapeutic effect and toxicological studies of BMI administered via IV injection into 4T1 tumor‐bearing mice. a) Schematic illustration of immunotherapeutic effect studies in vivo. Tumor‐bearing mice were tail vein injected with PBS, BM, BI, and BMI at determined dose (25 µg of IPI549 or 100 µg of Mn per mouse) every 2 days during medication. b) Tumor growth curves of mice after IV injection of different formulations (*n* = 5). Significance between the treatment groups and control group during the medication treatment (0–18 d) was calculated using unpaired two‐tailed Student's *t*‐test. *P < 0.05, **P < 0.01, ***P < 0.001. c) Survival rates of tumor‐bearing mice receiving various treatments (*n* = 5). d) H&E and TUNEL analyses of 4T1 tumor tissue sections harvested at the time point 18 d from mice receiving various treatments. e) H&E assay on liver and kidney tissues harvested at 18 d from various treatment groups. Scale bar: 100 µm. f–i) Serum levels of alanine transaminase (ALT), aspartate transaminase (AST), creatinine (CR), and blood urea nitrogen (BUN) in mice measured at 18 d during the course of treatment. H&E stained sections of the j) lungs and k) livers collected from 4T1 tumor‐bearing mice after drug withdrawal (i.e., at 26 d in Figure 4a, *n* = 5).

The immunotherapy was initiated by tail vein injection of BM, BI, and BMI solutions at an administration schedule of one injection every 2 d for 18 d. The tumor grew quickly in mice receiving PBS and BM to reach over 16 and 14 times of the initial volumes respectively, which indicated the ineffective tumor growth inhibition of hypoxia alleviation without concurrent PI3Kγ inhibition (**Figure** 4b). On the contrary, both BI and BMI exhibited obvious tumor growth inhibition. Moreover, BMI displayed even much stronger tumor growth inhibition than BI. Upon BMI treatment, tumor growth was nearly completely inhibited over the course of 18 d nanoregulator treatment. More excitingly, the BMI group and BI group displayed obviously different tumor re‐growth rates and metastatic potentials after ending medication (Figure 4b,j,k; Figure S8, Supporting Information). That is, the BMI group showed much slower tumor re‐growth and meanwhile much less metastasis to liver and lung.

Consistently, both BI and BMI greatly prolonged the survival time of animals, whereas BM almost showed no therapeutic effect like PBS (Figure 4c). These data clearly demonstrated that IPI549 delivered by the nanoregulators dominated the immunotherapy. Still, the codelivered MnO_2_ enhanced the immunotherapeutic effect by alleviating hypoxia of TME. We have demonstrated that BMI accumulated in tumor sites via EPR effect would disassemble/decompose in acidic TME. Consequently, the encapsulated IPI59 was released to exert immunotherapeutic effect, and meanwhile reduction of MnO_2_ resulted in O_2_ generation to relieve hypoxic condition which was reported to improve immunotherapeutic effect.^2^, ^3^ All animal groups showed no obvious weight loss (Figure S9, Supporting Information). Histological examinations showed no pathological change in major organs of mice receiving treatments (Figure 4e; Figure S10, Supporting Information), and the serum biochemical marks (ALT, AST, CR, and BUN) of mice receiving nanoregulator treatments were all within the normal ranges (Figure 4f–i).^50^, ^51^ These results demonstrated the low side effects of BM, BI, and BMI.

As shown in Figure 4d, BI and BMI showed much better therapeutic effects than PBS according to the remarkably decreased cancer cell number while elevated apoptosis level in the tumor tissue sections as measured by H&E and TUNEL assays. Furthermore, among the three therapeutic groups, BMI treatment resulted in the least cancer cells and meanwhile the highest apoptosis level in tumor tissue, indicating the best immunotherapeutic effect. Based on these results, we hypothesized that the alteration of hypoxic TME and inhibition of PI3Kγ on MDSC may have synergistically contributed to the best immunotherapeutic outcome of BMI. This notion will be further verified by exploring the effect of MnO_2_ and IPI549 codelivery on the tumor immune microenvironment (TIME).

### 2.6 Alleviation of Tumor Hypoxia and Inhibition of MDSC PI3Kγ

**Figure 5:**
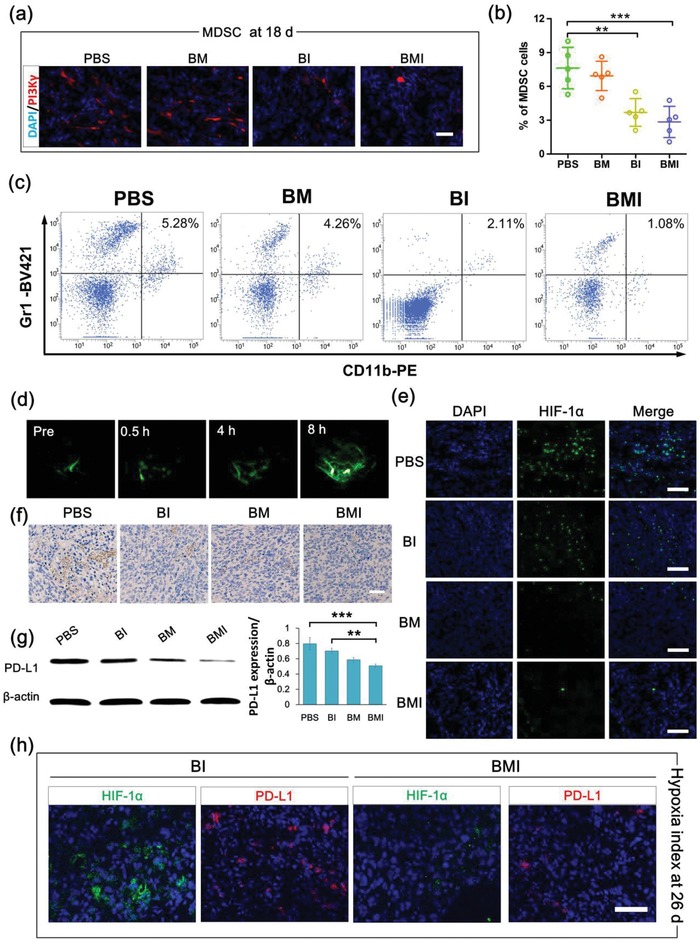
MnO_2_‐embedded nanoregulators inhibit PI3Kγ and alleviate tumor hypoxic microenvironment in vivo. a) Immunofluorescence images of tumor sections showing PI3Kγ inhibition after various treatments. The weakened immunofluorescence could be attributed to IPI549‐impeded antibody binding and decreased MDSCs infiltration. b) Ratio and c) representative flow cytometric plots of MDSC (CD11b^+^ Gr1^+^) in tumor tissue. ***P < 0.001, **P < 0.01. d) Representative 2D PA images of oxygenated Hb (λ = 850 nm) in 4T1 tumors before and after IV injection of BMI (25 µg of IPI549 or 10 µg of Mn per mouse). e) Immunofluorescence images of tumor slices excised from mice at 24 h after IV injection of various formulations. Tumor hypoxia was shown by anti‐HIF‐1α antibody staining. f) Immunohistochemical analysis of PD‐L1 expression in tumor slices collected from mice at 18 d during treatment (see Figure 4a for treatment schedule). g) Western blot analysis of PD‐L1 protein in 4T1 tumors collected from mice at 18 d during treatment. Scale bar: 100 µm. ***P < 0.001, **P < 0.01. h) Immunofluorescence assays displaying HIF‐1α and PD‐L1 expressions in tumor sections from mice after drug withdrawal.

Solid tumors commonly feature abnormal angiogenesis, which leads to sharp consumption of nutrition and oxygen, and meanwhile the accompanied secretion of H_2_O_2_ and H^+^ to further accelerate hypoxia and angiogenesis.^27^ Hypoxia of cancerous tissues has been known as a critical unfavorable factor for various cancer therapeutic means including immunotherapy,^18^, ^19^ and thus alleviation of the hypoxic conditions in TME was vital for tumor therapy. The effect of MnO_2_‐embeded nanoregulators on tumor hypoxia after their IV injection into BALB/c mice bearing subcutaneous 4T1 tumor was assessed by photoacoustic (PA) imaging, based on the 850 nm optical absorption of oxygenated hemoglobin (Hb) indicative of the O_2_ concentration in tumor blood vessels. BMI showed a sustained elevation of oxygenated Hb after IV injection (**Figure** 5d), which can be explained by the aforementioned gradual tumor accumulation of nanoregulator to allow a sustained O_2_ generation via MnO_2_ reaction at tumor acidic and H_2_O_2_‐enriched conditions.

It is well known that HIF‐1α is a pivotal indicator of tumor hypoxic status related to tumor cell proliferation, apoptosis, angiogenesis, anaerobic glycolysis, and hypoxia‐associated therapeutic resistance. Thus, tumor progression can be suppressed by down‐regulation of HIF‐1α.^52^, ^53^ Immunofluorescence staining assay was carried out to investigate the HIF‐1α regulation of tail‐vein injected BMI in the 4T1 tumor‐bearing animal model. The rapid proliferation and premature development of blood vessels in tumor sites lead to O_2_ deficiency and overexpressed HIF‐1α (Figure S11, Supporting Information). Compared to that from the PBS‐treated mice, the tumor tissues from mice receiving BMI and BM showed obviously decreased tumor hypoxia area, as evidenced by the greatly weakened hypoxic signals revealed by HIF‐1α antibody staining (Figure 5e). The results validated that the MnO_2_‐embedded nanoregulators (BMI or BM) were delivered to tumor hypoxic region, and then reacted with H_2_O_2_ and H^+^ to efficiently release O_2_. Therefore, HIF‐1α was suppressed as a result of hypoxia alleviation.

Although immune checkpoint blocking antibodies against CTLA‐4 or PD‐1/PD‐L1 have displayed incredible immunotherapeutic effects in some cancer types,^5^ tumors may adopt multiple mechanisms mediating immune resistance. Growing evidences are suggesting that the high infiltration of immune‐suppressive myeloid cells play a key role in poor prognosis and resistance to ICB.^6^, ^7^ In recent researches,^8^, ^15^ small molecular PI3Kγ inhibitors such as IPI549 showed great potential to restore the ICB sensitivity of tumors with high level of suppressive myeloid cell infiltration. Here, the PI3Kγ inhibition capabilities of BM and BMI on tumor tissues were evaluated by immunostaining and flow cytometric assays (Figure 5a–c). As PI3Kγ is highly expressed on MDSCs,^15^, ^16^, ^17^ tumor tissue from mice receiving PBS treatment displayed high level of PI3Kγ immunostaining. In contrast, treatments with PI3Kγ nanoregulators (BMI and BI) carrying IPI549 led to marked decrease in PI3Kγ immunostaining in tumor tissues. BM without carrying IPI549 did not show appreciable reduction in the immunostaining of PI3Kγ. In the flow cytometric assay, the ratio of CD11b^+^Gr1^+^ MDSC in tumor tissue was lowered in the three treatment groups (BM, BI, and BMI), with BMI showing the most remarkable suppression. Apparently, IPI549 released from the nanoregulators in the TME, as shown in Scheme 1, could effectively bind to the PI3Kγ membrane protein, which in turn could affect the binding of fluorescently labeled PI3Kγ antibody. Moreover, the decreased number of CD11b^+^Gr1^+^ MDSC might have contributed to the weakened PI3Kγ immunofluorescence staining as well. These results suggested that reshaping the TIME solely by alleviating hypoxia via MnO_2_ delivery had little effect on the PI3Kγ expression. However, MnO_2_ codelivered with IPI549 did contribute to a better PI3Kγ inhibition as BMI appeared more potent than BI in weakening the PI3Kγ immunostaining.

### 2.7 Evidences of Nanoregulator‐Reshaped TIME for Effective Immunotherapy

Systematic studies were carried out to evaluate the effects of nanoregulator treatments on TIME reshaping, including the PD‐L1 down‐regulation, TAMs polarization, effector T cell activation, and T_reg_ cell suppression. Furthermore, as the BMI group showed more significant inhibition in tumor re‐growth and metastasis than the BI group after ending nanoregulator administration, we also investigated the immune memory effects in the two groups of mice surviving immunotherapy in order to gain more insight into the underlying causes for the differences in post‐medication anticancer effects.

Previous studies have shown that the overexpressed HIF‐1α in hypoxic TME binds to the HRE in the PD‐L1 proximal promoter, which leads to significantly upregulated expression of PD‐L1 on MDSC, macrophages, dendritic cells, and tumor cells.^20^ Therefore, we expected MnO_2_ to exert a PD‐L1 down‐regulation effect via oxygenation to suppress HIF‐1α. At the time point terminating medication (18 d), the expression of PD‐L1 in tumor tissue of mice was clearly down‐regulated due to the treatments of BI, BM, and BMI in comparison with PBS (Figure 5f,g). Especially, BMI treatment showed the most significant suppression of PD‐L1 expression, most likely due to a synergistic effect of MDSC‐suppression by IPI549 and alleviation of hypoxic condition by MnO_2_.^20^, ^54^ Then, the redevelopment of hypoxic TME after drug withdrawal was investigated by determining the expressions of HIF‐1α and PD‐L1. As shown in Figure 5h, mice of the BMI group showed significantly lower expressions of HIF‐1α and PD‐L1 than mice of the BI group. Apparently, the rapid tumor re‐growth in mice receiving BI rebuilt a hypoxic microenvironment inside tumor, which in turn might have promoted the tumor re‐growth after drug withdrawal. On the contrary, BMI treated mice showing much slower tumor growth was hard to restore the hypoxic and PD‐L1‐overexpressed TME. On the other hand, the BMI treatment prevented metastasis of 4T1 tumors to lung and liver much more efficiently than the BI treatment (Figure 4j,k; Figure S8, Supporting Information), which may partially owe to the suppressed redevelopment of hypoxic TME in the BMI treatment group. These notions were in line with previous reports. For examples, hypoxia influences the fate of disseminated tumor cells (DTCs) by up‐regulating key dormancy (NR2F1, DEC2, p27) and hypoxia (GLUT1, HIF‐1α) genes.^55^ Furthermore, hypoxic microenvironment of solid tumor imprinted subpopulations of DTCs to become an NR2F1‐dependent dormant state, which may escape therapies and exacerbate incurable metastasis.^56^

**Figure 6:**
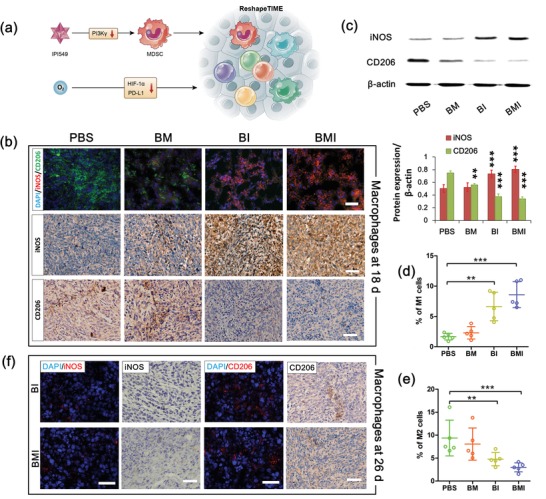
Nanoregulator directed the polarization of TAMs from an immunosuppressive M2‐like phenotype (marker: CD206) to a pro‐inflammatory M1‐like phenotype (marker: iNOS). a) Schematic illustration of reshaping tumor immune microenvironment (TIME) by pluripotent nanoregulator. b) Immunofluorescence or immunohistochemical assays showing in situ expressions of macrophage markers in 4T1 tumor from mice after various treatments at 18 d. Scale bar: 100 µm. c) Western blotting showing expressions of iNOS and CD206 at 18 d (see Figure 4a for the treatment schedule) in 4T1 tumor from mice receiving various treatments. d) M1 (CD11b^+^ F4/80^+^ CD16/32^+^) and e) M2 (CD11b^+^ F4/80^+^ CD206^+^) phenotype macrophage ratios in tumor tissue determined by flow cytometry at 18 d after various treatments. f) Immunofluorescence and immunohistochemical assays displaying expressions of macrophage markers in tumor sections collected from mice after drug withdrawal. Brown stains indicated CD206 or iNOS protein while blue stains showed nuclei in immunohistochemical assay. **P < 0.01, ***P < 0.001.

The effect of reshaping TIME to induce TAMs polarization upon PI3Kγ inhibition was also evaluated in order to further understand the in vivo performance of nanoregulators. MDSC, an immature cell, is the precursor cell of M1 and M2 macrophages. The hypoxic TME promotes the differentiation of MDSC into immune suppressive TAMs, which is known as a process directed by HIF‐1α.^57^ As shown in **Figure** 6 and Figure S12 (Supporting Information), PI3Kγ inhibition with the BMI and BI in vivo resulted in the polarization of TAMs into a M1‐like phenotype rather than a M2‐like phenotype in tumor tissue according to the protein biomarker immunostaining and Western blotting. In other words, the iNOS as a typical biomarker for the M1 phenotype was significantly up‐regulated, whereas CD206 as a typical biomarker for the M2 phenotype was significantly down‐regulated upon the BI and BMI treatments.^27^ Moreover, BMI appeared more potent in promoting such polarization toward M1‐polarized TAMs. At one week after ending nanoregulator treatment, only the expression of iNOS (M1 marker) in the BMI and BI treatment groups was restored to the levels equivalent to that in the PBS group. However, both the BI and BMI treated mice still showed decreased expressions of CD206 (M2 marker) as compared to the PBS group. It is well known that the M1‐polarized macrophage is proinflammatory, whereas the M2‐polarized one is anti‐inflammatory (i.e., immunosuppressive).^8^ Thus, switching TAMs into M1 phenotype has emerged as a key strategy for cancer immunotherapy in recent years.^58^ Our results evidenced the great potential of BMI in reshaping the TIME for TAMs polarization in favor of immunotherapy.

**Figure 7:**
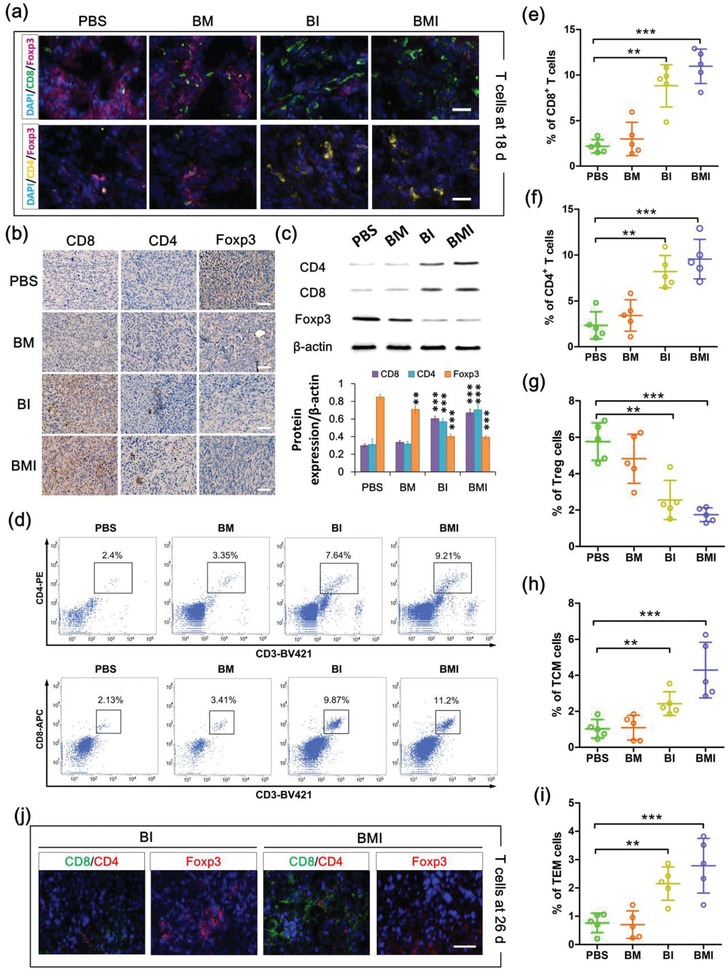
PI3Kγ inhibition by BMI regulated immune cells to reshape tumor immune microenvironment. a) Immunofluorescence and b) immunohistochemical assays showing expressions of CD8 (cytotoxic T cell marker), Foxp3 (T_reg_ cell marker) and CD4 (T helper cell marker) in tumors at the time point 18 d of treatment scheme (see Figure 4a). Scale bar: 100 µm. c) Western blotting showing expressions of CD4, CD8, and Foxp3 in tumors from mice receiving various treatments at 18 d. d) Representative flow cytometric plots showing CD4^+^ T cell and CD8^+^ T cells ratios in tumor tissue after various treatments. e–i) Flow cytometry showing proportions of T cell subsets in tumor tissues at 18 d. j) Immunofluorescence and immunohistochemical assays showing markers of T cell subsets in tumor‐bearing mice after drug withdrawal. ***P < 0.001, **P < 0.01.

Finally, the effect of nanoregulators on T lymphocytes via reshaping TIME in tumor tissue was evaluated. The TME can promote conversion of MDSCs to nonspecific suppressor cells through up‐regulating enzymes involving in the metabolism of L‐arginine. These enzymes (iNOS and arginase I) play key roles in T cell suppression.^59^, ^60^ MDSC not only inhibits the function of CD4^+^ T cells by reducing the extracellular content of L‐arginine via arginine synthase, but also inhibits the function of CD8^+^ T cells via iNOS and arginine synthase. Such T cell suppression was aggravated under hypoxia, which is mediated by the HIF‐1α transcription factor.^54^ Previous studies have shown that inactivation of PI3Kγ on macrophage promotes an immunostimulatory transcriptional program activating CD8^+^ T cells,^15^ which is highly cytotoxic to tumor cells. Moreover, the CD4^+^ T cells are considered “helper” cells which could recognize tumor cells and then interact with CD8^+^ T cells for tumor inhibition.^61^ On the contrary, the regulatory T cells (T_reg_) are able to suppress antitumor immunity.^31^, ^62^ Therefore, the effects of our nanoregulators on these functional cells in tumor tissue were explored. Excitingly, in comparison with tumor tissue of animals receiving PBS, tumor tissues of animals receiving BM, BI, and BMI treatments all showed reduced T_reg_ cells (**Figure** 7; Figure S13, Supporting Information). Furthermore, significantly enhanced CD8^+^ and CD4^+^ T cell infiltration was observed in tumor tissues of mice receiving BMI or BI, although the enhanced CD8^+^ and CD4^+^ T cell infiltration in tumor tissue of mice receiving BM was much less obvious (Figure 7; Figure S13, Supporting Information).

Previous studies have shown that memory T cells, classifiable into central memory T (TCM) cells and effector memory T (TEM) cells,^63^ may transform to effector T cells for persistent immunological effect after drug withdrawal.^64^, ^65^, ^66^ We utilized flow cytometry to quantify the TCM and TEM cells in tumor tissues of mice receiving BMI, BI, BM, and PBS at the time point (18 d) ending nanoregulator treatment (Figure 7h,i). The percentages of both TEM and TCM cells were much higher in mice treated with IPI549‐containing nanoregulators (BMI and BI) than that in mice receiving PBS or BM. Furthermore, the BMI‐treated mice with the highest percentage of memory T cells also displayed the most abundant CD8^+^ T cells and CD4^+^ T helper cells in tumor at one week after drug withdrawal, which implied that the prominent long‐term inhibition of tumor re‐growth and metastasis in the BMI group was also due to elevated numbers of memory T cells (Figure 7j). In addition, it was noted that the Treg cells were persistently suppressed in the BMI group, which may have also contributed to a better immunotherapeutic effect. Nevertheless, the underlying cause for persistent T_reg_ cell suppression was not clear yet.

The above results strongly demonstrated that the nanoregulator activated the immune response to tumor mainly via IPI549 binding to PI3Kγ on MDSCs, and the distinct feature of BMI releasing payload in response to TME apparently had a critical contribution to the IPI549 action. Moreover, the codelivered MnO_2_ alleviated hypoxia to result in down‐regulation of the immunosuppressive PD‐L1 on tumor cells, which could be another underlying cause for the remarkable immunotherapeutic effect of our nanoregulator possessing multiple functions including TAMs polarization, T lymphocyte regulation, PD‐L1 down‐regulation, and tumor‐specific MRI. Our results also showed that the animals treated with multifunctional nanoregulator effectively reserved the reshaped TIME toward persistent inhibition of tumor re‐growth and metastasis.

## 3 Conclusions

BSA‐based nanoregulator incorporating MnO_2_ and PI3Kγ inhibitor IPI549 was developed to reshape TIME. Encapsulation of MnO_2_ made the nanoregulator highly sensitive to tumor acidic and H_2_O_2_‐enriched environment, leading to oxygenation and meanwhile nanoregulator collapse for rapid IPI549 release. Consequently, the produced Mn^2+^ enabled a tumor‐specific MR imaging, the hypoxia alleviation down‐regulated the expression of immunosuppressive PD‐L1, and the binding of released IPI549 to PI3Kγ on MDSC resulted in the M1‐polarization of TAMs and activation of tumor‐suppressive T‐lymphocytes. This work shows the great potential of a novel TIME nanoregulator to mediate highly effective MRI‐visible immunotherapy of cancer.

## 4 Experimental Section

*Materials*: Bovine serum albumin, MnCl_2_, nile red, and 1,1′‐dioctadecyl‐3,3,3′,3′‐tetramethyl indotricarbocyanine iodide (DiR) were obtained from Sigma‐Aldrich. IPI549 was supplied by MCE (Shanghai, China). HIF‐1α antibody was purchased from Santa Cruz Biotechnology (California, USA). Anti‐CD31 antibody, anti‐PI3K p110 gamma antibody, and other antibodies for immunofluorescence staining, immunohistochemical staining, and WB analysis were obtained from Abcam (Shanghai, China).

*Nanoregulator Synthesis and Characterization*: A series of MnO_2_‐containing nanoregulators were prepared by one‐step synthesis method. Briefly, 2 mL of BSA solution (10 mg mL^−1^) was mixed with 0.1 mL of MnCl_2_ (12.6 mg mL^−1^), and the pH value of the mixture was adjusted to 11 using NaOH. Then, the mixture was left for 2 h at 37 °C for the formation BSA‐MnO_2_ (BM).^28^

For the drug‐loaded formulations, BSA‐MnO_2_‐IPI549 (BMI), drug was encapsulated by directly mixing the solution of IPI549 (50 µL of 10 × 10^−3^ m) with the solution containing BSA and MnCl_2_. Nanoparticles loaded with NR rather than IPI549 were also prepared by mixing the solution of NR (100 µL of 2 mg mL^−1^) with the solution containing BSA and MnCl_2_.

Nanoparticles loaded with NR (BN) and IPI549 (BI) were prepared by simply mixing the BSA solution with NR or IPI549, respectively, without adding MnCl_2_. 2 mL of BSA solution (10 mg mL^−1^) was mixed with NR (100 µL of 2 mg mL^−1^) or IPI549 (50 µL of 10 × 10^−3^ m), and the pH value of the mixture was adjusted to 11 using NaOH. Then, the mixture was left for 2 h at 37 °C for the formation of BN or BI.

The UV–vis absorption spectra were recorded using the PerkinElmer UV750 spectrophotometer. The Mn quantification was carried out using inductive coupled plasma‐mass spectrometry (ICP‐MS, iCAP RQ, Thermo Fisher Scientific, USA). NR was quantified by fluorescence spectrophotometer (RF5301, Shimadzu, Japan) at Ex = 488 nm and Em = 580 nm. The size and morphology of the nanoparticles were analyzed by dynamic light scattering and transmission electron microscopy.

The dissolved O_2_ was determined with a portable dissolved oxygen meter (Taizhou Leici Instrument Equipment Co. Ltd., Taizhou, China). The O_2_‐free water was obtained by bubbling N_2_ for 10 min, and then BMI or BM was added as O_2_ supplier. Afterward, the mixed solutions were adjusted to pH 6 and/or 100 × 10^−6^ m of H_2_O_2_, and the O_2_ concentrations were recorded.

*Relaxivity Measurement of BMI*: The magnetic relaxation properties of BMI at different conditions were measured. BMI was diluted with neutral PBS (pH 7.4), acidic PBS (pH 6.8), neutral PBS + H_2_O_2_ (50 × 10^−6^ m), and acidic PBS (pH 6.8) + H_2_O_2_ (50 × 10^−6^ m) respectively in a 96‐well plate, then scanned by using a clinical 3.0 T MRI scanner (Philips, Achieva, Philips Medical Systems, Best, The Netherlands) with an 8‐channal sense knee coil. The acquisition sequences included T1WI, T2WI, mixed inversion‐recovery spin echo T1‐map sequence, and single‐section multispin‐echo T2‐map sequence. The detailed acquisition parameters were described previously.^67^ The *r*_2_ and *r*_1_ relaxivities of BMI were calculated from the slope of the linear plots of *r*_2_ and *r*_1_ relaxation rates (s^−1^) versus Mn concentration (× 10^−3^ m) by using linear least‐squares regression analysis. The magnetic relaxation properties of MnCl_2_ were measured as a positive control.

*Cell Experiments*: Murine breast cancer 4T1 cells were cultured at 37 °C in a humidified atmosphere of 5% CO_2_ for detection of cytotoxicity. The cell viability was assessed through CCK‐8 assay. Briefly, cells were seeded in a 96‐well plate at a density of 5 × 10^3^ cells per well in the presence of 100 µL of RPMI‐1640 culture medium. BM, BI, and BMI were added at different concentrations, and cells were incubated for another 24 h. 10 µL of CCK‐8 solution (Kumamoto, Japan) was added to each well to incubate the cells for 2 h at 37 °C. CCK‐8 assay was carried out according to the manufacturer's instructions. All experiments were performed in triplicate.

After incubation with 100 µg mL^−1^ of BM, BI, BMI, or PBS as a control for 24 h, 4T1 cells were harvested and underwent the Annexin V‐FITC/ propidium iodide (PI) double staining for flow cytometry analysis (FACScalibur; BD, Mountain View, CA, USA) to reveal cell apoptosis investigation. For cell cycle analysis, the 4T1 cells were harvested and fixed in ice‐cooled 70% ethanol for 24 h, resuspended in PBS containing RNase A (50 mg mL^−1^) and incubated for 30 min at 37 °C, incubated with propidium iodide (PI, 100 mg mL^−1^) for 30 min at 4°C, and analyzed for DNA contents using flow cytometry.

Immunofluorescence assay was performed to evaluate PD‐L1 expression in 4T1 tumor cells. After incubation with different formulations for 24 h, the 4T1 cells were subjected to immunostaining with the anti‐PD‐L1 antibody. After sequential incubation with primary antibody at 4 °C overnight and secondary antibody at 37 °C for 1 h, the cells were stained with DAPI and finally imaged under CLSM.

*In Vivo Imaging and Therapeutic Assay*: BALB/c female mice aged 4–6 weeks were purchased from Vital River Co. Ltd. (Beijing, China) and used following protocols approved by Animal Care and Use Committee of the Sun Yat‐sen University. A total of 1 × 10^7^ 4T1 cells were subcutaneously injected into right flank of each mouse. DiR was loaded together with drug into BMI particles for fluorescence imaging. When the tumors grew to detectable volume, 100 µL of DiR‐containing BMI was intravenously injected at a dose of 1 mg DiR kg^−1^ body weight. The fluorescence images were captured on in vivo imaging system (Carestream IS 4000, USA) at determined post‐injection time intervals. At determined time points, mice were euthanized and ex vivo fluorescence imaging of main organs and tumors were conducted.

To detect the dynamic changes of MR signal intensity, in vivo MRI study was performed on a clinical 3.0‐T MR system (Achieva; Philips Medical Systems) with a 50 × 50 mm 4‐channel phased array rat coil (Zhongzhi Medical Technologies, Suzhou, China). MR scans were performed at 0.5, 2, 6, 12, 24, 48, and 72 h after tail vein injection of BMI at a dose of 5 mg Mn kg^−1^ body weight. The acquisition sequences included axial and coronal FSE T1WI: TR/TE (500/20 ms), Flip angel (20°), NSA (3), FOV (50 × 50 mm), matrix (256 × 256), and section thickness/gap (1.0/0.0 mm). The T1WI signal intensity and contrast enhancement ratio of tumors on T1WI were measured and calculated using ImageJ (NIH, Bethesda, MD, USA). For each slice, the signal intensity of tumors was manually outlined by using the region of interest (ROI). The contrast enhancement ratio of tumor was calculated as a percentage of the signal intensity enhanced at different time points after injection compared with that at the preinjection. To compare the contrast‐enhancement of BMI with that of ionic manganese (MnCl_2_) as a positive control, the dynamic changes of MR signal intensity after intravenous/intratumoral injections of MnCl_2_ at a dose of 5 mg Mn kg^−1^ body weight were measured.

Before and at 2, 12, and 72 h after intravenous/intratumoral injections of BMI and MnCl_2_, the brain, heart, lung, liver, spleen, and kidney of 3 randomly chosen mice in each group were harvested, weighted, and then dissolved in the mixture of 30% hydrogen peroxide solution and nitric acid for 7 days. Then, the tissues were digested using a microwave digesting system. The content of Mn in different organs was measured by ICP‐MS (iCAP RQ, Thermo Fisher Scientific, USA).

For therapeutic assays, when the tumors growth reached ≈200 mm^3^, BALB/c mice were randomly divided into 4 groups, and intravenously injected once every 2 days with PBS, BM, BI, and BMI at determined dose (25 µg of IPI549 or 100 µg of Mn per mouse). Tumor sizes were daily measured by a caliper and the tumor volumes were calculated by (length × width^2^)/2. The mouse body weights were also recorded. The mice were euthanized after therapeutic experiments or when the tumor volumes reached 3000 mm^3^, and then tumor tissue and main organs (heart, liver, spleen, lung, and kidney) were dissected and further used for histology and biochemical analysis.

*In Vivo Biodistribution and Toxicological Studies*: The concentration of ionic Mn in major organs and tumors was measured to evaluate in vivo distribution and systemic retention of the nanoregulators. The tumor‐bearing mice were euthanized at determined time points after IV or i.t. injection of BMI or MnCl_2_. Tissues were then harvested, weighed, and digested by nitric acid. The amount of Mn in solutions was quantified using ICP‐MS after dilution and filtration. The Mn content was expressed as the mass of Mn in one gram of individual organ.

To further determine the toxic and side effects of nanoregulators, serum samples of tumor‐bearing mice were withdrawn after a treatment course for serum biochemical tests. ALT and AST were used to evaluate liver injury while CR and BUN were utilized to assess renal function.

*Ex Vivo Immunofluorescence, Immunohistochemical, and Western Blot Analysis*: At predetermined time points during the immunotherapy process, mice were sacrificed and tumor tissues were embedded in optimum cutting temperature compound (OCT) and kept frozen at −80 °C. 5 µm sections were cut and subjected to immunostaining with the primary antibody. After incubation at 4 °C overnight, the secondary antibody was added to detect primary antibody, followed by sequential incubation and finally scanning with CLSM. To obtain the immunohistochemically stained samples, the collected tumors from mice treated with different formulations were fixed in 4% paraformaldehyde, embedded in paraffin, and then sliced into 2 µm sections. After deparaffinating and immunostaining with primary and secondary antibodies, the sections were observed by an optical microscope. The 4T1 tumors were collected and homogenized for Western blot analysis after different treatments. Protein contents were determined by BCA kits. Proteins were loaded for SDS‐PAGE and transferred to polyvinylidene difluoride (PVDF) membranes.

To evaluate the hypoxia relief effect of MnO_2_‐containing nanoregulators, rabbit polyclonoal HIF‐1α antibody and rabbit anti‐CD31 antibody as the primary antibody were used for staining of HIF‐1α and blood vessels, respectively. Anti‐PD‐L1 antibody was used to evaluate the expression of immunosuppressive PD‐L1 on the surface of both tumor cells and MDSCs. PI3Kγ was stained to investigate the selectivity of IPI549. The M1 and M2 phenotypes (with markers of iNOS and CD206, respectively) of MDSC were also estimated. Further, the markers of CD8^+^/CD4^+^ T cells, and Foxp3, the marker of T_reg_ cells were used to indicate the capability of tested formulation to regulate TIME.

*Ex vivo Flow Cytometry Analysis of Immune Cells*: At 14 d after treatment in each group, the tumors were collected and homogenized within PBS (pH 7.4) to obtain single‐cell suspensions. Cells were first stained with live/dead staining, then stained with the corresponding antibodies of anti‐CD11b‐PE, anti‐F4/80‐BV421, anti‐CD206‐AlexaFluor 647, anti‐CD16/32‐FITC, anti‐CD4‐PE, anti‐CD3‐BV421, anti‐CD8‐APC, anti‐CD25‐FITC, anti‐Foxp3‐Cy5.5, anti‐CD4‐FITC, anti‐CD62L‐PE, and anti‐Gr1‐BV421. Except that anti‐Foxp3‐Cy5.5 was purchased from eBioscience (San Diego, CA, USA), all the other antibodies were purchased from BD Biosciences (San Jose, CA, USA). Single‐cell suspensions were analyzed on a FACScan flow cytometer (FACScalibur, BD, USA). Gating was first performed to exclude the dead cells and adhesive cells. CD4^+^ cells were further gated as CD3^+^ CD4^+^; CD8^+^ T cells gated as CD3^+^ CD8^+^; macrophages were further gated as CD11b^+^ F4/80^+^, then subgated as M1 macrophages (CD11b^+^ F4/80^+^ CD16/32^+^ CD206^−^) and M2 macrophages (CD11b^+^ F4/80^+^ CD206^+^ CD16/32^−^); MDSC were further gated as CD11b^+^ Gr1^+^; regulatory T cells were further gated as CD4^+^ CD25^+^ Foxp3^+^. Memory T cells were further gated as CD3^+^ CD8^+^, then subgated as central memory T cells (TCM, CD3^+^ CD8^+^ CD62L^+^ CD44^+^), and effector memory T cells (TEM, CD3^+^ CD8^+^ CD62L^−^ CD44^+^). The percentages of positively cells relative to total cell number were calculated.

*Statistical Analysis*: Data analyzed using one‐factor analysis of variance (SPSS software, version 13.0, SPSS Inc.) were expressed as mean ± SD. *P* < 0.05 was considered of statistical significance. All statistical tests were two‐sided.

## Conflict of Interest

The authors declare no conflict of interest.

## Supporting information

Supplementary
