# Supplementary material for: Multifunctional Nanoregulator Reshapes Immune Microenvironment and Enhances Immune Memory for Tumor Immunotherapy
Source: Adv Sci (Weinh). 2019 Jun 17;6(16):1900037. doi: 10.1002/advs.201900037 (PMC6702652; doi:10.1002/advs.201900037)
Supplement: Supplementary file 1 — Supplementary [file ADVS-6-1900037-s001.pdf]

Copyright WILEY-VCH Verlag GmbH & Co. KGaA, 69469 Weinheim, Germany, 2019.

ADVANCED SCIENCE Open Access

Supporting Information

for *Adv. Sci.,* DOI: 10.1002/advs.201900037

Multifunctional Nanoregulator Reshapes Immune Microenvironment and Enhances Immune Memory for Tumor Immunotherapy

Meng Yu, Xiaohui Duan, Yujun Cai, Fang Zhang, Shuqi Jiang, Shisong Han, Jun Shen, and Xintao Shuai**

Copyright WILEY-VCH Verlag GmbH & Co. KGaA, 69469 Weinheim, Germany, 2019.

Supporting Information

Multifunctional Nanoregulator Reshapes Immune Microenvironment and Enhances Immune Memory for Tumor Immunotherapy

Meng Yu, Xiaohui Duan, Yujun Cai, Fang Zhang, Shuqi Jiang, Shisong Han, Jun Shen, and Xintao Shuai**

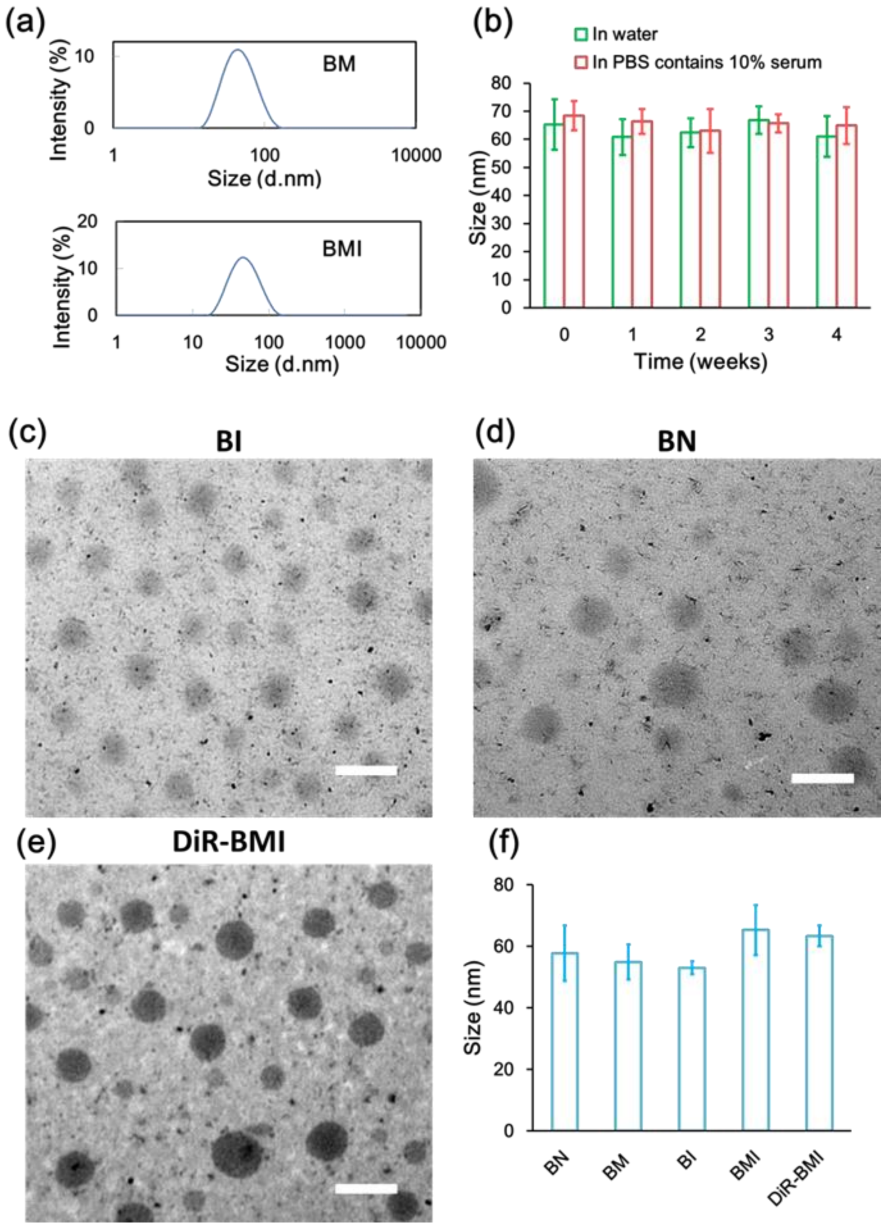

Figure S1. (a) Hydrodynamic diameters of MnO_2-embedded nanoparticles before (BM) and after (BMI) encapsulation of therapeutic drug IPI549. (b) BMI was stable in aqueous solution and serum-containing medium over 4 weeks. (c-e) TEM images of BI, BN, and DiR-labeled

BMI. Scale bar: 200 nm. (f) Average sizes of different nanoparticles (n = 3).

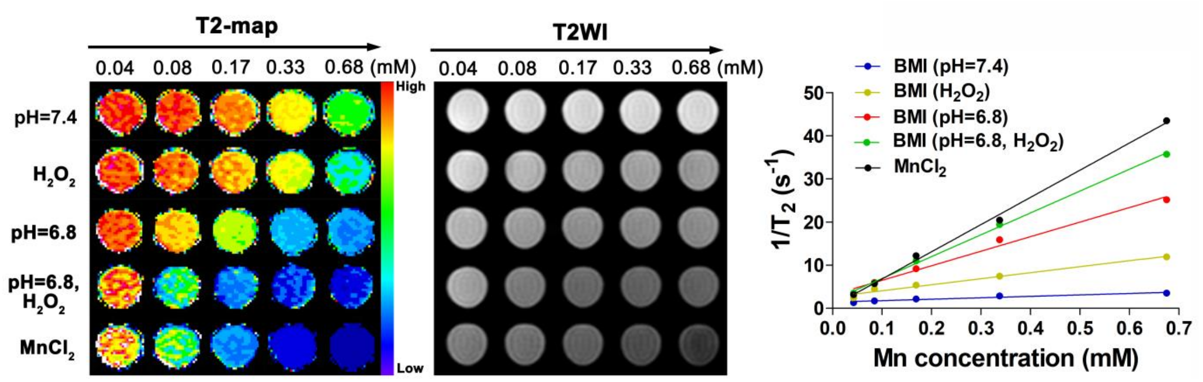

Figure S2. T2-map and T2WI (a), measurements for longitudinal relaxivities (*r2*) (b) of BMI at various conditions. The magnetic relaxation properties of $MnCl_2$ as a positive control were measured as well.

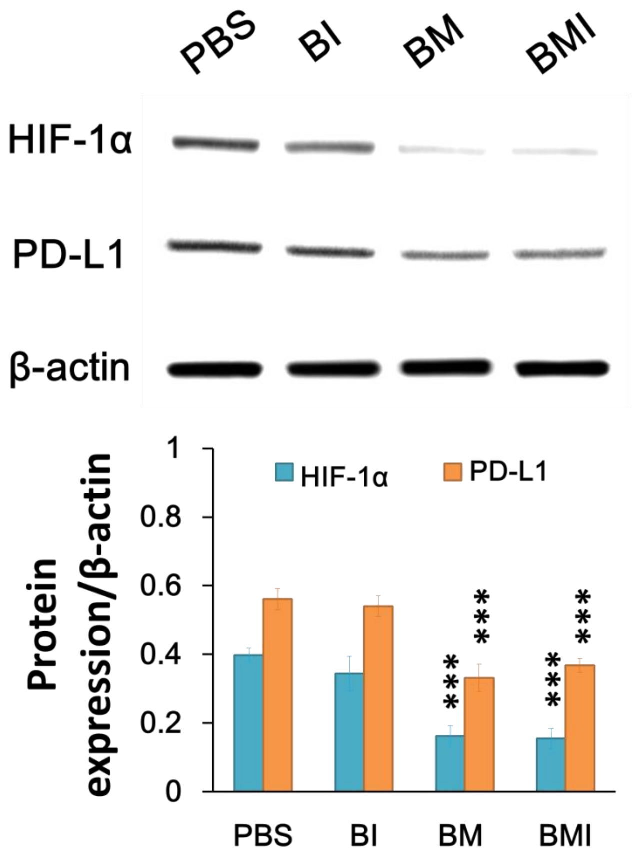

Figure S3. Western blotting showing expressions of HIF-1α and PD-L1 in 4T1 cells after incubation for 24 h with different formulations (n = 3). ***P < 0.001.

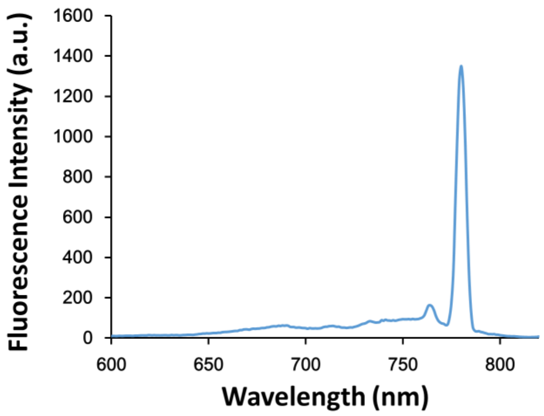

Figure S4. Fluorescence spectra of DiR-labeled BMI nanoparticles (Ex = 764 nm and Em = 780 nm).

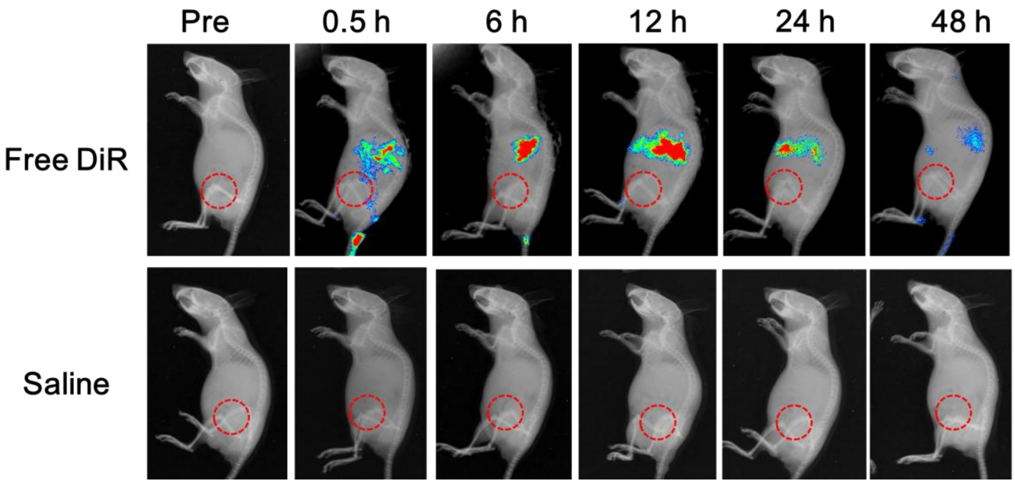

Figure S5. Representative *in vivo* fluorescence imaging of 4T1 tumor-bearing mice intravenously injected with free DiR (8 mg kg^{-1})and saline at different time points.

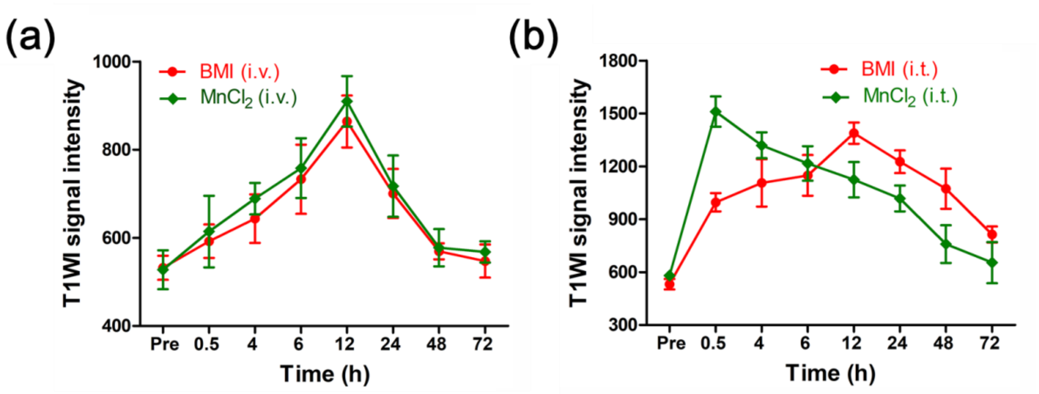

Figure S6. A comparison of the T1WI signal intensity of tumors after i.v. (a) and i.t. (b) administration of BMI and $MnCl_2$ (n = 3).

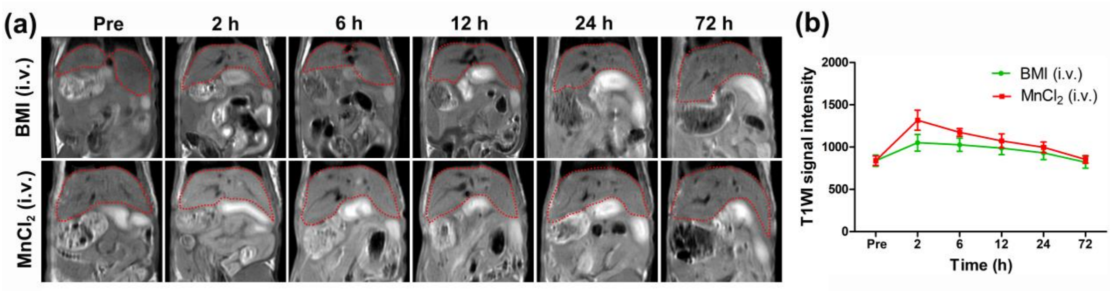

Figure S7. T2-weighted MR imaging (a) and signal intensities (b) of liver after i.v. administration of BMI (5 mg Mn kg^{-1}) and $MnCl_2$ at different time points on T1WI (n = 3).

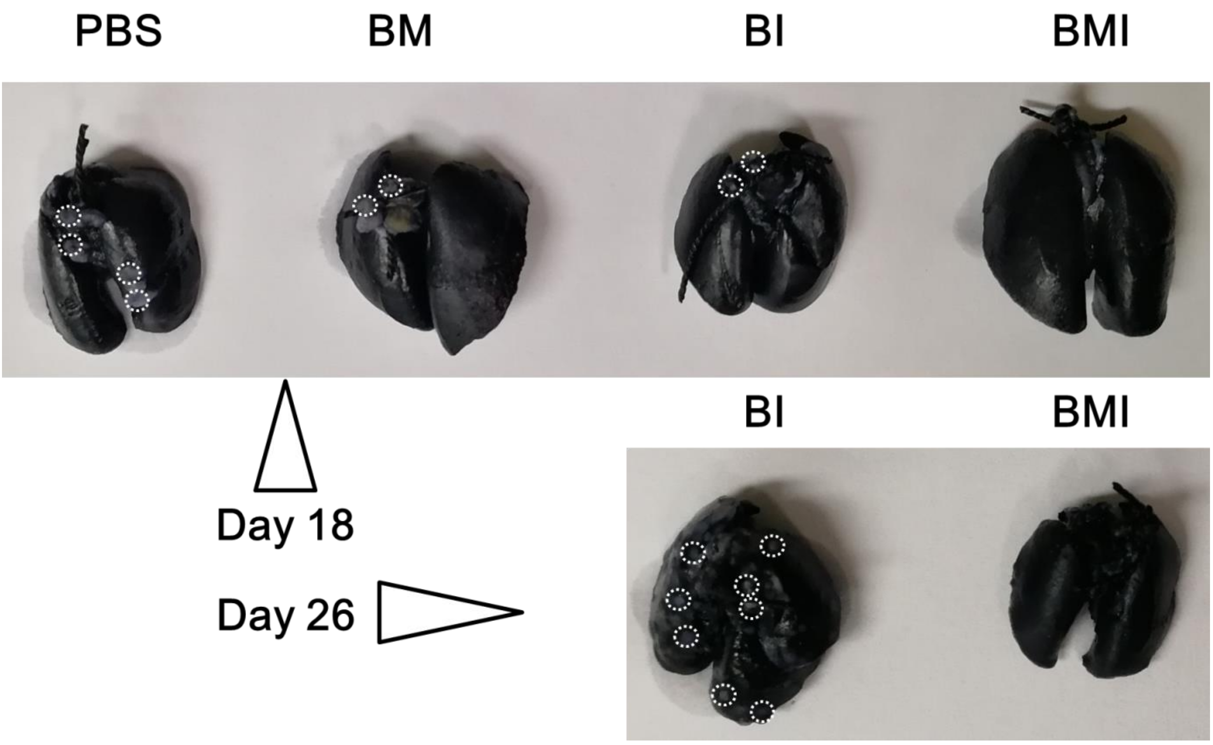

Figure S8. Representative photographs of lung tissues during the treatment course. The white dotted line indicated the lung metastatic foci.

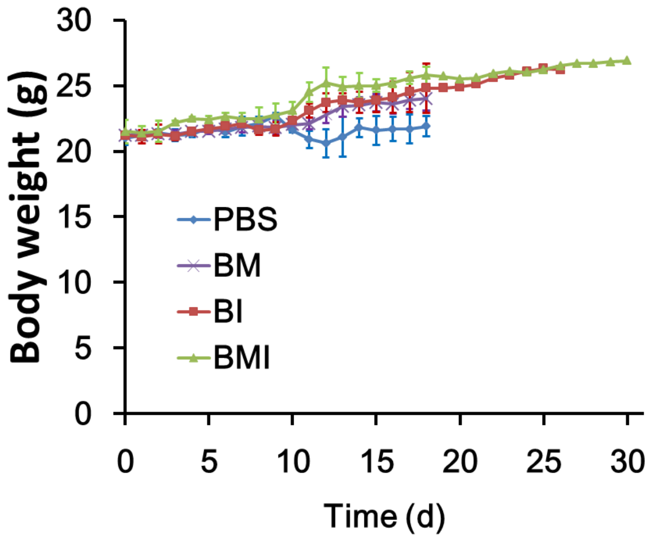

Figure S9. Changes of body weights of tumor-bearing mice receiving various treatments (n = 5).

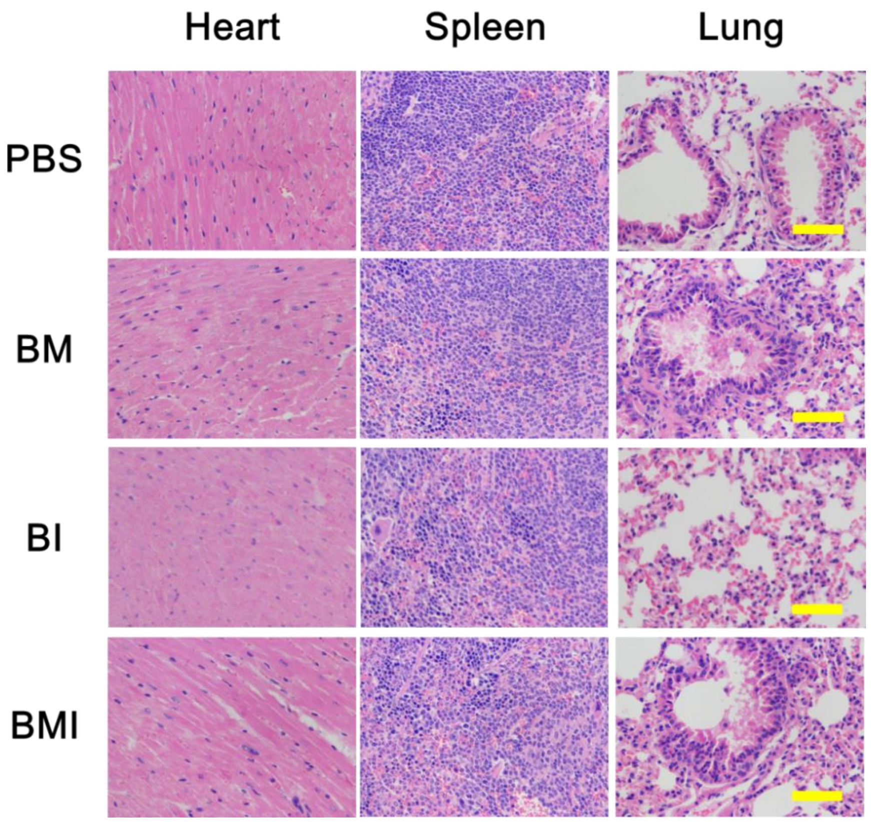

Figure S10. H&E staining for heart, spleen, and lung tissues from mice receiving various treatments at 18 d of treatment. Scale bar: 100 μm.

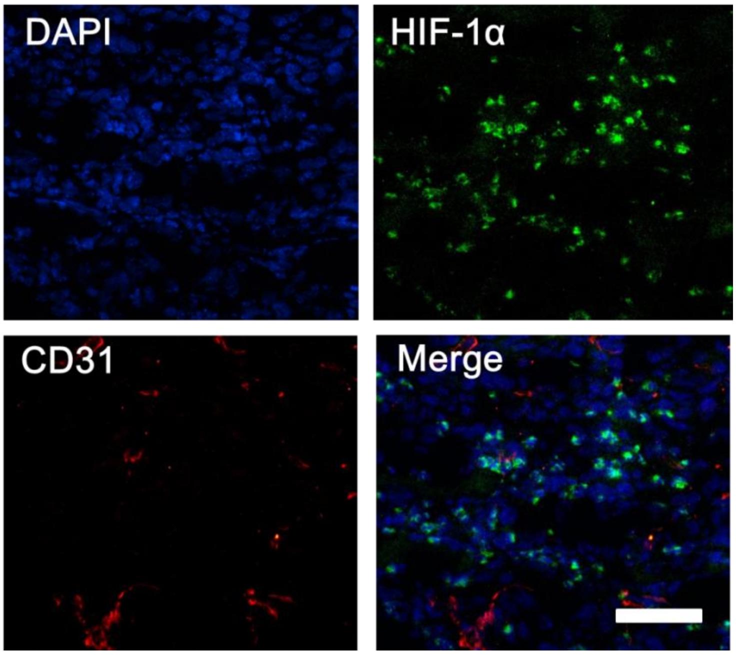

Figure S11. Immunofluorescence images of tumor slices from mice 24 h after i.v. injection of PBS. Tumor hypoxia was determined by anti-HIF-1α antibody staining. CD31 was analyzed for endothelial cells of tumor neovasculature. Scale bar: 100 μm.

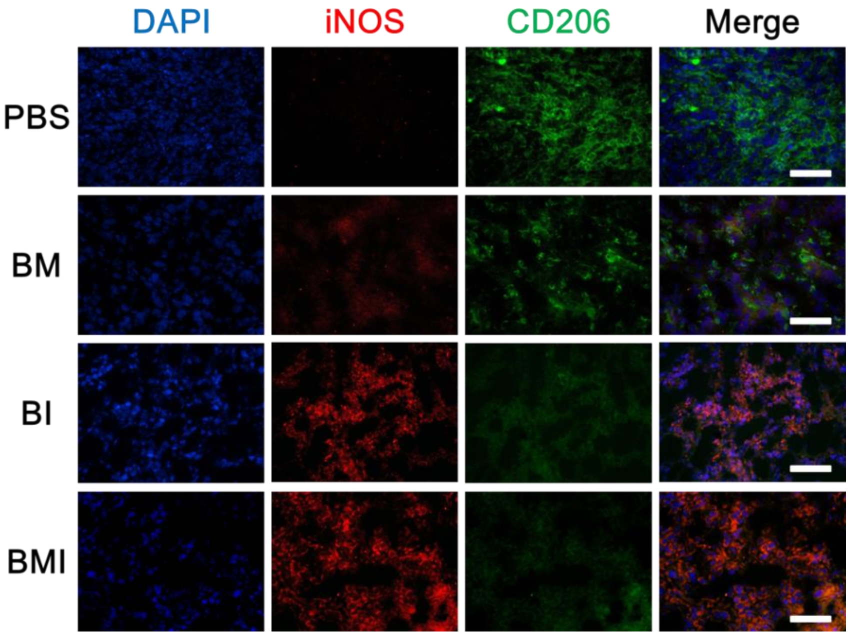

Figure S12. Immunofluorescence analysis for M1 and M2 markers in tumor tissue of mice receiving various treatments at 18 d of treatment. Scale bar: 100 μm.

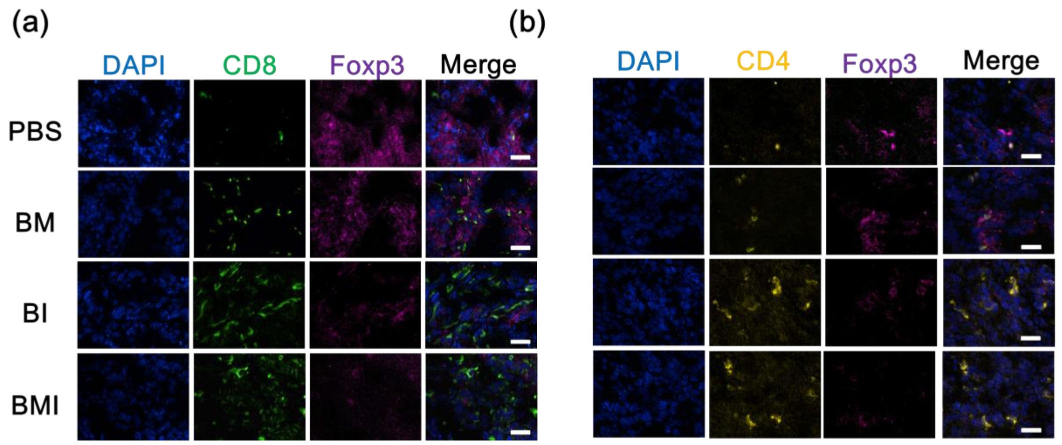

Figure S13. Immunofluorescence analysis for expressions of CD8 (a) and CD4 (b) over Foxp3 at 18 d of treatment (also see Figure 7a only showing the merged images). Scale bar: 100 μm.
